# Early Fault Diagnosis Method for Batch Process Based on Local Time Window Standardization and Trend Analysis

**DOI:** 10.3390/s21238075

**Published:** 2021-12-02

**Authors:** Yuman Yao, Yiyang Dai, Wenjia Luo

**Affiliations:** 1 College of Chemistry and Chemical Engineering, Southwest Petroleum University, Chengdu 610500, China; 201921000234@stu.swpu.edu.cn (Y.Y.); luowenjia@swpu.edn.cn (W.L.); 2 School of Chemical Engineering, Sichuan University, Chengdu 610065, China

**Keywords:** QTA, batch processes, incipient fault detection

## Abstract

The products of a batch process have high economic value. Meanwhile, a batch process involves complex chemicals and equipment. The variability of its operation leads to a high failure rate. Therefore, early fault diagnosis of batch processes is of great significance. Usually, the available information of the sensor data in batch processing is obscured by its noise. The multistage variation of data results in poor diagnostic performance. This paper constructed a standardized method to enlarge fault information as well as a batch fault diagnosis method based on trend analysis. First, an adaptive standardization based on the time window was created; second, utilizing quadratic fitting, we extracted a data trend under the window; third, a new trend recognition method based on the Euclidean distance calculation principle was composed. The method was verified in penicillin fermentation. We constructed two test datasets: one based on an existing batch, and one based on an unknown batch. The average diagnostic rate of each group was 100% and 87.5%; the mean diagnosis time was the same; 0.2083 h. Compared with traditional fault diagnosis methods, this algorithm has better fault diagnosis ability and feature extraction ability.

## 1. Introduction

Batch processing is extensively utilized in modern production fields such as food, materials, chemicals, and pharmaceuticals [1]. The features between batch data make processes difficult to control, presenting multi-stage characteristics in the time dimension, and a strong correlation in the variable dimension [2]. Introducing fault diagnosis technology into the batch process can effectively guarantee personnel safety and reduce economic loss. Different batches of data differ at the same time due to subtle differences in their environment, human operations, and initial conditions. As a result, the diagnosis performance of traditional fault diagnosis methods decreases. At the same time, noise often covers weak fault information in the early stage of the fault, leading to delayed detection and misdiagnosis problems [3]. So, the research of early fault diagnosis technology in batch processes is crucial for the safe operation of the chemical plant.

Early fault diagnosis methods for batch processes are divided into mechanism-based, knowledge-based, and data-based methods [4,5,6]. It is hard to build diagnostic models based on physical and chemical mechanisms [7], so the research on fault diagnosis methods tends to be the latter two. Data-based fault diagnosis techniques used in batch processing are mainly multivariate statistical methods and deep learning methods [8,9]. The former mainly calculate statistics and thresholds to fault detection. Hoo, K. converted 3D batch data into 2D data for the first time, and then input it into the principal component analysis (PCA) process for batch process fault detection [10]. According to this data conversion method, the multi-way partial least square (MPLS) [11] and other common batch fault detection methods have been successfully developed. However, the traditional multivariate statistical early fault diagnosis method still has the problems of false positives and poor diagnosis timeliness [12]. Peipei Cai et al. [13] proposed the multi-block probability correlation kernel principal component analysis (KPCA) method to measure the change of probability distribution caused by a small offset, reducing the detection time. Yihao Qin et al. [14] combined sliding window technology with traditional statistical detection methods. They used the improved rank-one correction method to perform a recursive calculation of singular value decomposition, reducing the computational complexity and false positive rate. He et al. invented a multivariate statistical method based on the detrending and denoising techniques, increasing the difference between fault trends and reducing the influence of noise [15]. Deep learning belongs to a black-box model [16]. On the premise of the sufficient data, it has a good fault identification effect and feature extraction ability for highly nonlinear processes [17]. Therefore, multiple and multivariate statistical methods are combined to identify fault types; for example, the convolutional neural network (CNN) [18,19], dynamic Bayesian network (DBN) [20,21], long short term memory (LSTM) [22,23], etc.

The data-based fault diagnosis method can automatically mine the data relationship, having certain universality. However, it is difficult to process the data, as it requires intricate theoretical knowledge [24]. Qualitative trend analysis (QTA), a semi-quantitative method, can retain more information by combining qualitative knowledge mining with data relations [25]. In 1990, Cheung and Stephanopoulos defined the qualitative trend language [26,27]. In 1991, Janusz and Venkatasubramanian characterized different trends with the magnitude and sign of first- and second-order differentials [28]. In 1992, Konstantinov and Yoshida used a polynomial fitting method to reason about the temporal shapes of the process variables [29]. In 1994, Bakshi and Stephanopouslos used the decision tree method to match trends [30]. Thus, a complete QTA method with language definition, trend extraction, and trend matching is formed. The traditional QTA method will lose a large amount of useful information because there are only seven basic elements [31]. In addition, the helpful information of industrial data is concealed in the environmental noise. Meanwhile, different fault degrees have diverse noise distributions [32]. So, the original QTA has disadvantages in diagnosis. Early researchers used fuzzy theory to fuzzy match the trend of the knowledge base to reduce the influence of noise [33]. Later, they achieved more robust information base building and matching by bridging different data-driven methods [34,35]. Those methods sacrifice the training and computation time of the algorithm. QTA based on fuzzy theory has an ability to resist noise. However, it performs poor in multi-fault type recognition [33].

To expand the early fault information, improve the effect of trend analysis in batch fault diagnosis, and ensure the smooth operation of the process, the local adaptive standardization method based on time window and QTA with pattern recognition is proposed—called the LAS-QTA method. First, a new sliding window-based local adaptive standardization is constructed to solve the problem where normal conditions cannot be unified due to the differences between different normal batches. Second, the trend representation in the traditional QTA method is improved to obtain more trend information. Then, a new trend matching method based on Euclidean distance was created to avoid the error of trend matching caused by the difference of a few variables. Final, this study constructed a complete framework for early fault diagnosis based on local adaptive standardization (LAS) and trend analysis.

The rest of this paper is organized as follows: Section 2 mainly explains the relevant principles and describes the novel fault diagnosis method designed; Section 3 introduces the application and discussion of this method in a penicillin fermentation process; and Section 4 presents the conclusions and orientations for future research.

## 2. Methods and Improvements

### 2.1. Local Adaptive Standardization

Because of the difference in magnitude between variables, some intelligent methods need to standardize the data before being used. Since the traditional standardization method will reduce the separability of data after processing multi-modal data, Ma et al. [36] proposed the local neighborhood standardization (LNS) method to standardize. It calculates the mean and standard deviation in the local domain of data. However, when there is no similar data in the database, the use effect of subsequent algorithms becomes worse. In 2020, Wu et al. [37] proposed a new LNS method based on the time window, achieving good results in multi-batch problems. The formulas are as follows:

(1) $$z_{i}=\frac{x_{i}-\mathrm{mean}\left(w_{i}\right)}{\mathrm{gmstd}\left(X\right)},$$

(2) $$\mathrm{gmstd}\left(X\right)=\sqrt{\frac{n_{1}{\left(std\left(X_{1}\right)\right)}^{2}+\ldots +n_{p}{\left(std\left(X_{p}\right)\right)}^{2}}{n_{1}+\ldots +n_{p}}},$$

where $x_{i}$ is the sample to be normalized, $z_{i}$ is the sample after normalization, and $n_{i}\left(i=1\sim p\right)$ is the number of samples in mode i of the training data set. The $\mathrm{mean}\left(w_{i}\right)$ denotes the mean vector of the sample in the local moving window, and $std\left(X_{i}\right)$ denotes the standard value of the samples in mode i.

In the actual process, normal data under a new schema or a new batch may not exist in the historical database. So, we modified Formulas (1) and (2) to obtain an adaptive local normalization method based on the time window in this paper. The standardized formula is as follows:

(3) $$z_{i}=\frac{x_{i}-\mathrm{mean}\left(w_{i}\right)}{std\left(w_{i}\right)},$$

where $w_{i}$ is the time series corresponding to the time window where $x_{i}$ is located. The smaller the length of the time window is, the more the accuracy of the standard deviation will reduce, and the more the impact of noise will amplify. As long as the subsequent fault diagnosis method can reduce the influence of noise, the normalization method can effectively retain useful information and achieve the goal of normalizing the distribution of the same type of data.

### 2.2. Qualitative Trend Analysis

**Figure 1:**
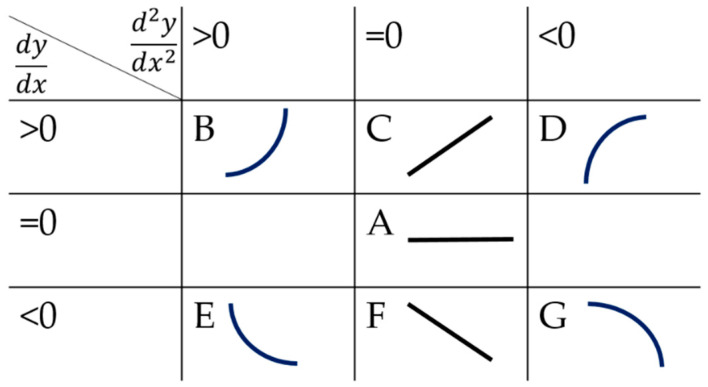
QTA knowledge base based on derivative. A~G are seven different primitives derived from the relationship between the derivatives and zero.

The QTA methods roughly consists of two steps: trend extraction and trend analysis, and in further detail it consists of three parts: the language to represent trends, the method to extract trends and core information, and the classification method (trend matching) [28]. The first thing is to determine what is the extracted information based on the task. Then, the appropriate trend extraction method needs to be selected according to the data. Next, the method-based QTA needs to analyze the extracted trend data to determine the language representing the trend. Finally, the last step of the method is building the classification method, such as the most commonly QTA method. It extracts the positive and negative of the first and second derivatives of the fitted curve as the trend language through the least square fitting of the data. Then, QTA knowledge bases are built. Finally, it determines the category by comparing the knowledge base, as shown in Figure 1. This approach has difficulties in distinguishing trends that have nuances in angles and positions due to the characteristics of the batch process data, making it less effective in early batch fault diagnosis.

**Figure 2:**
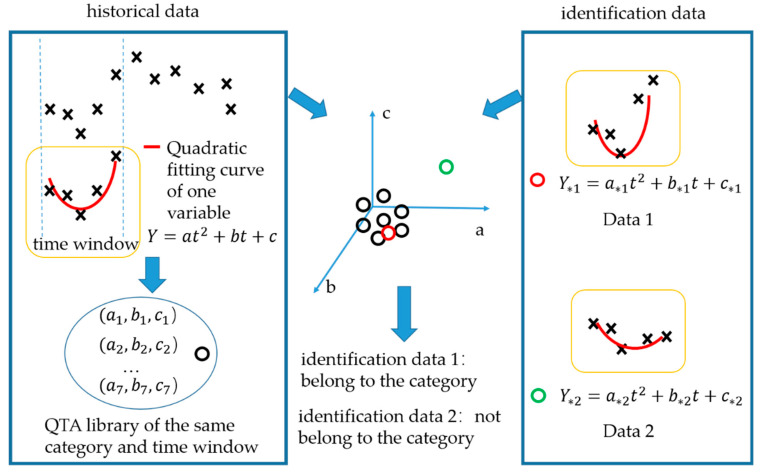
The basic principle of the proposed method.

This paper proposed a new language of trend expression and a new trend matching method based on the original QTA idea to solve the problem. The basic principle is shown in Figure 2. The new method carried out the unitary quadratic fitting on the data in the time window, and used the index (*a*, *b*, *c*) in the function to represent the trend, which makes the extracted information larger. Meanwhile, we used the spatial distance to match the trends, reducing the nuances of the same model that have been amplified by the introduction of time windows. For example, in the schematic diagram, the distances between data 1 and historical data in the coordinates are less than the distances between the historical data. So, data 1 is considered to belong to the category of historical data. The data cluster formed by identification data 2 and historical data can be perfectly separated. So, it is not considered to belong to this category. The specific identification method of the distance can be set according to the concrete situation.

Under different normal conditions, the trend of a few variables may differ significantly. We combine the coefficient (*a*, *b*, *c*) of all variables to reduce false positives caused by this difference. Then, the original time window data is converted into the following vector: [a1, b1, c1, a2, b2, c2, a3, b3, c3, …… an, bn, cn]. The space of the calculating distance is changed from three dimensions to 3*n* dimensions. Converting low-dimensional data into a high-dimensional space is easier to cluster and segment. This principle has been proven in the invention and subsequent use of KPCA. So, the fault detection (binary classification) and fault identification (multi-classification) tasks can be accomplished theoretically by combining appropriate pattern recognition way in the proposed method.

### 2.3. Fault Diagnosis Model

**Figure 3:**
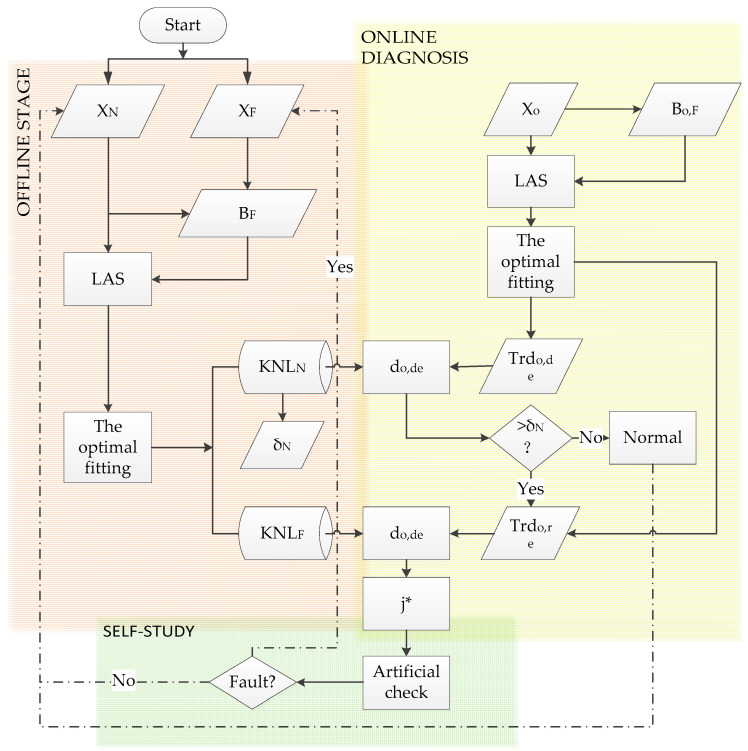
The fault diagnosis framework based on QTA and LAS.

Combined with the theory of the method, a new fault diagnosis method with functions of offline preparation, online diagnosis, and self-learning is proposed, called LAS-QTA. The diagnosis flow chart is shown in Figure 3.

#### 2.3.1. Offline Stage

Step 1: Use Formula (3) to standardize the historical normal data *X_N_*.

Step 2: Calculate historical fault deviation data *B_F_* by using the historical normal data and historical fault data. The form of historical fault data *X_F_* is as follows to ensure that the data in the first window has only one failure data point:

(4) $$X_{F}=\left[x_{F,t_{start}},x_{F,t_{start}+1},\ldots \ldots ,x_{F,t_{end}}\right],$$

(5) $$t_{start}=t_{intro}-w_{time}+1,$$

where $t_{intro}$ is the time of introduction of the fault; $w_{time}$ is the width of the time window; $t_{end}$ is the time of fault sampling point. Then, the calculation formula of the historical fault deviation matrix $B_{F}$ is as follows:

(6) $$B_{F}=X_{F}-\left[x_{N,t_{start}},x_{N,t_{start}+1},\ldots \ldots ,x_{N,t_{end}}\right],$$

where $x_{N}$ is the normal sample closest to $X_{F}$.

Step 3: Use Formula (3) to standardize the historical fault deviation data $B_{F}$.

Step 4: Optimal fitting of unary quadratic equation is carried out for each data sample under each time window. The principles are as follows:

(7) $$f_{k}\left(x\right)=a_{k}x^{2}+b_{k}x+c_{k},$$

(8) $$\varepsilon_{k}=\sum_{i=1}^{m}{\left[f\left(x_{i}\right)-y_{i}\right]}^{2},$$

(9) $$f_{*}\left(x\right)=a_{*}x^{2}+b_{*}x+c_{*},\;*={\mathrm{argmin}\varepsilon }_{k},$$

where $f_{*}\left(x\right)$ is the optimal quadratic equation of one variable; $f_{k}\left(x\right)$ is the k-th quadratic equation of one variable; $\varepsilon_{k}$ is the square error corresponding to the kth fitting quadratic equation of one variable; a, b, c are the fitting coefficients of quadratic equations of one variable.

Step 5: Establish the QTA knowledge base for subsequent online diagnosis, and its data structure is as follows:

(10) $$\mathrm{KNL}=\left[{\mathrm{Knl}}_{w1},{\mathrm{Knl}}_{w2},\ldots \ldots ,{\mathrm{Knl}}_{ws}\right],$$

where ${\mathrm{Knl}}_{wi}$ represents the knowledge base corresponding to the i-th time window, *s* denotes the number of time windows. Its data form is as follows:

(11) $${\mathrm{Knl}}_{wi}=\left[Trd_{w,1},Trd_{w,2},\ldots \ldots \right],$$

(12) $$Trd=\left[a_{*,1},b_{*,1},c_{*,1},a_{*,2},b_{*,2},c_{*,2},a_{*,3},b_{*,3},c_{*,3},\ldots \ldots ,a_{*,n},b_{*,n},c_{*,n}\right],$$

where n is the number of features. The Trd is called the trend information vector. The$\;a_{*,i}$, $b_{*,i}$, $c_{*,i}$ are the coefficients of the i-th variable of the unitary quadratic equation obtained by Equations (7)–(9) for optimal fitting.

Step 6: Calculate the threshold of the normal QTA knowledge base, the principle is as follows:

(13) $$\delta_{w}=\max d_{w,i,j},$$

(14) $$d_{w,i,j}=Trd_{w,i}\cdot Trd_{w,j},$$

where the i-th normal sample in window w dot the j-th’s. The result is $d_{w,i,j}$. The max value of the $d_{w,i,j}$ is considered as the threshold of the normal in window w. That is to say that the threshold of the normal QTA knowledge base is a vector.

#### 2.3.2. Online Diagnosis Stage

Step 1: Extract online data, which is in the form of a time window data:

(15) $$X_{o}=\left[x_{t-w_{time}+1},x_{t-w_{time}+2},\ldots \ldots ,x_{t}\right],$$

where $X_{o}$ is the online data; $w_{time}$ is width of time window; $x_{t}$ is the data of *t* time; $x_{t-w_{time}+2}$ is the data of *t*-*w_time_*_+2_ time. So $X_{o}$ is a matrix composed of data from $w_{time}$ sampling points.

Step 2: Use Formula (3) to standardize the online data. Fit the data to get $Trd_{o,de}$.

Step 3: Calculate the Euclidean distance with the historical normal QTA library of the corresponding time window:

(16) $$d_{o,de}=\min\left(Trd_{o,de}\cdot Trd_{N,wt,k}\right),Trd_{N,wt,k}\epsilon {\mathrm{Knl}}_{N,wt},$$

where $Trd_{o,de}$ is the trend information vector of $X_{o}$. $Trd_{N,wt,k}$ is the *k*th trend information vector in wt window of the normal knowledge base.

Compare with δ, if $d_{o,de}\leq \delta$, then the data is considered as normal and stored in the corresponding historical database. Otherwise, the data is considered as being faulty, and we proceed with the following steps.

Step 4: To obtain online fault deviation data, the calculation method is as follows:

(17) $$B_{o,F}=X_{o}-X_{N*},$$

(18) $$X_{N*}=X_{N,k_{*}},\;k_{*}=\mathrm{argmin}\left(Trd_{o,de}\cdot Trd_{n,wt,k}\right),$$

where $B_{o,F}$ is the online fault deviation data; $X_{N*}$ is the normal data matrix in the history library closest to the online data. The assessment criteria are the Euclidean distance.

Step 5: Use Formula (3) to preprocess $B_{o,F}$; use Formulas (7)–(9) to obtain the recognition trend $Trd_{o,re}$.

Step 6: Calculate the Euclidean distance with the different historical fault QTA library in the corresponding time windows $d_{o,re,j}$:

(19) $$d_{o,re,j}=\min\left(Trd_{o,re}\cdot Trd_{Fj,wt,k}\right),Trd_{Fj,wt,k}\epsilon {\mathrm{Knl}}_{Fj,wt},$$

where $Trd_{o,re}$ is the trend information vector of $B_{o,F}$. $Trd_{Fj,wt,k}$ is the kth trend information vector in wt window of fault j knowledge base.

Step 7: Judge the fault type according to the following formula:

(20) $$j^{*}=argmin\left(d_{o,re,j}\right).$$

Step 8: Report the result.

#### 2.3.3. Self-Study Stage

If $d_{o,de}\leq \delta$, store the data in a historical database in Step 3 of the online diagnosis; feedback fault type results to the operator for verification and correction, and save the data to the corresponding fault database after correction before processing Step 8. If the result of the manual check is the new fault, expand the history library on top of the original one and run the offline part again.

## 3. Application to the Fed-Batch Fermentation of Penicillin Process

### 3.1. Process Description

**Figure 4:**
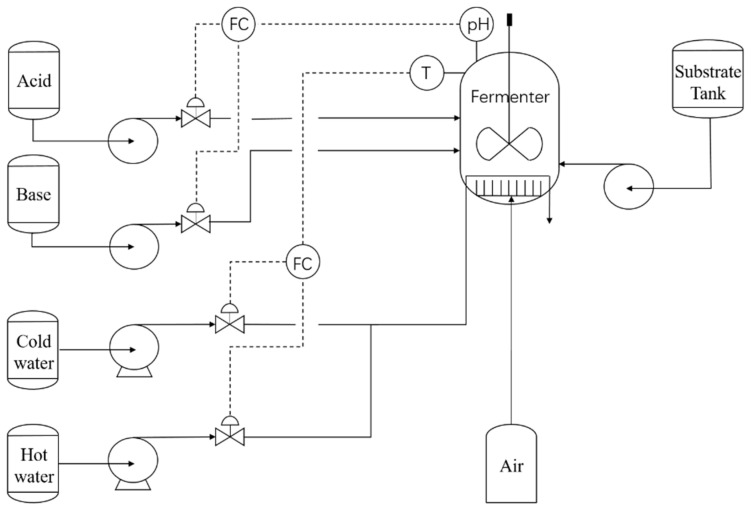
The process of penicillin fermentation.

Penicillin fermentation is the most important process in the production of penicillin, and its technological process is shown in Figure 4.

The main reaction of the process takes place in a fermenter with a stirrer. Two PID controllers—pH and temperature—ensure the stability and efficiency of the reaction. Fully ferment through the sufficient mixed contact of air and fermentation at a set pH and temperature value. The whole fermentation process divides into three stages: thallus growth stage, penicillin synthesis stage, and thallus autolysis stage. The data of the penicillin synthesis stage are highly nonlinear, which is the stage most prone to fault. The used data in this paper came from PenSim V2.0 software. PenSim2.0 is the software developed by the Cinar research group to simulate the penicillin fermentation process [38]. It is the main simulation software for batch processing due to its close degree of data to the real situation and simple operation. The software can simulate the following fault types: aeration rate step increasing, aeration rate step decreasing, agitator power step increasing, agitator power step decreasing, substrate feed rate step increasing, and substrate feed rate step decreasing. There are 17 variables in the model. We select the following variables as diagnostic objects: the flow of air, dissolved oxygen concentration, real volume of fermentation liquid, carbon dioxide concentration, pH value, and cold water flow, which are denoted by A, B, C, D, E, and F, respectively.

**Figure 5:**
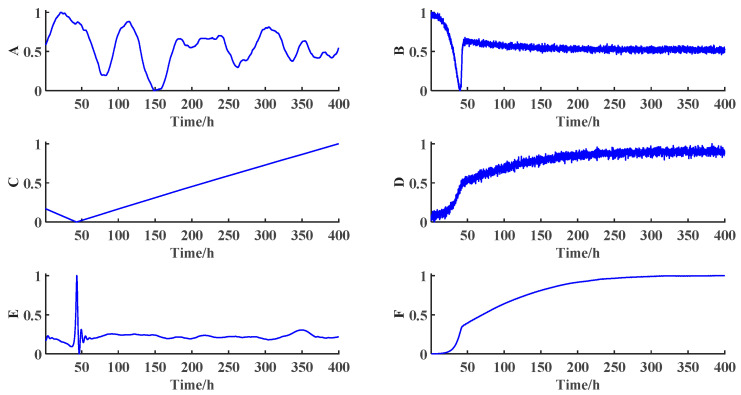
The diagnostic variables trend of a normal sample obtained by running PenSim based on the settings of batch 1. A: the flow of air; B: dissolved oxygen concentration; C: real volume of fermentation liquid; D: carbon dioxide concentration; E: pH value; F: cold water flow.

In this paper, the simulation time of each batch is 400 h and the sampling interval is 0.1 h. The initial set values of the normal batches used are shown in the Table 1. Batches 1 to 3 are used for offline preparation. The diagnostic variables trend of normal batch 1 is shown in Figure 5. The historical fault samples are mainly the fault data with 50% amplitude deviation ending at 400 h when normal batch 2 runs under set conditions for 70 h.

The test set of this paper has 24 groups of fault batches. They were obtained by running the software based on the settings of Batches 1 and 4. Each type of fault has four groups of samples with different amplitude. The specific situation is shown in Table 2 and Table 3.

### 3.2. Results and Discussion

The LAS-QTA fault diagnosis program was completed according to the steps of Section 2.3. The used data was generated from PenSim based on Table 1, Table 2 and Table 3. The diagnosis results are shown in Table 3. The table shows that when the corresponding normal condition is batch 4, samples 11, 12, and 17 were identified as other categories. It indicates that the method has the following disadvantages in fault identification: If there is no corresponding normal sample in the history database, the fault identification effect will be reduced. However, the proposed method has the same fault detection performance in two data sets.

The detection time distribution of different fault type samples is not equal in Table 4. Analysis of the reasons shows that faults 2, 3, 5, and 6 do not directly affect the collected sensor data, but rather indirectly. Additionally, the variable change caused by the fault is much smaller than other faults and there is a time lag problem. Therefore, the detection time of batches of fault types 1 and 4 is much shorter than that of other types of fault data. In conclusion, the established QTA method has certain adaptability and stronger fault diagnosis ability.

There may be a time dislocation between the detection time and the optimal diagnosis time. Therefore, this paper judged the fault type of the data in 10 time windows after the corresponding time when the fault was detected. We use the fault identification accuracy rate (FDA) as the evaluation index. Its calculation formula is as follows:

(21) $$\mathrm{FDA}=\frac{\mathrm{The}\;\mathrm{number}\;\mathrm{of}\;\mathrm{data}\;\mathrm{identify}\;\mathrm{correctly}\;}{\mathrm{Total}\;\mathrm{count}\;\mathrm{of}\;\mathrm{test}\;\mathrm{data}}.$$

**Figure 6:**
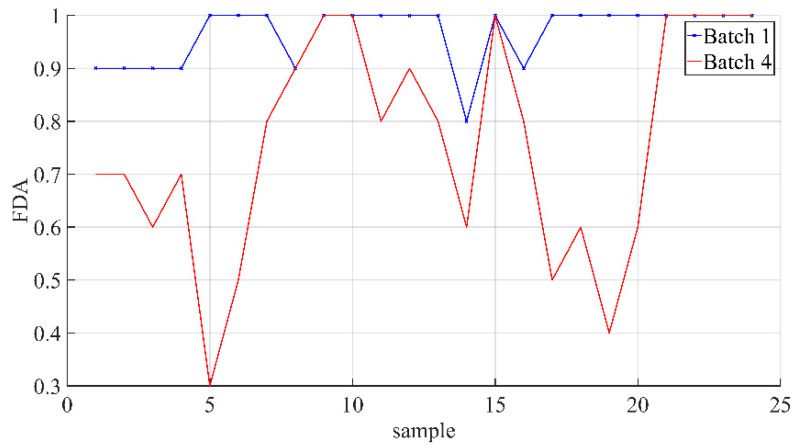
The relationship of the average diagnostic rate in 10 time windows under different normal conditions.

**Figure 7:**
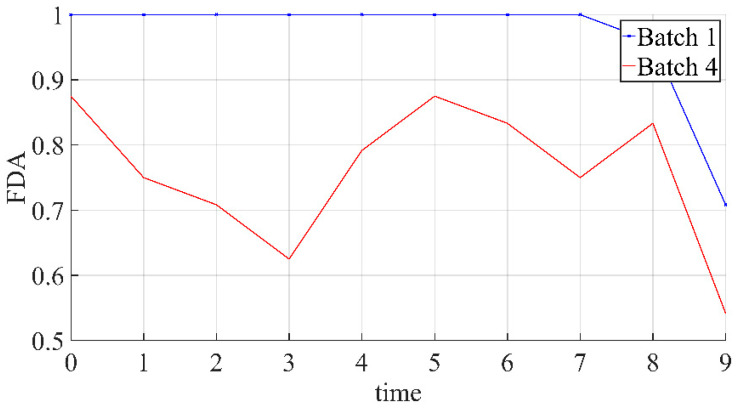
The relationship between the average diagnosis rate under different normal conditions in each time window.

The relationship between the average diagnostic rate of different samples in 10 time windows under different normal conditions is shown in Figure 6. The relationship between the average diagnosis rate under different normal conditions in each time window is shown in Figure 7.

Figure 6 shows that the robustness of this method is poor in the case that there is no corresponding normal batch in the historical QTA database. This indicates that the fault identification ability based on the proposed method is insufficient and needs to be improved in the future.

Figure 7 illustrates that the effectiveness of the method for fault identification changes over time. At the same time, when there is no corresponding normal sample in the history database, the diagnostic robustness in time is lower. There are two main reasons why the diagnosis rate of Batch 1 starts to decrease at 8-time points: first, the range of fault samples in the fault history database is 50%, and the diversity is low. Second, there is the problem that the trend difference degree of the error matrix of different faults will weaken over time. The randomness of the Batch 4 diagnosis rate curve is mainly related to the following reasons: the time of fault detection is the first time that the system considers that there is an unacceptable deviation from the normal situation. If this time lag exists in nature, then the fault deviation matrix of subsequent time does not match the corresponding deviation matrix. That is, the data difference between the formed fault deviation matrix and the real deviation matrix will change irregularly with the backward moving of the window.

Combining the two figures, it can be found that when there is a corresponding normal batch in the history database, the fault identification ability will change because of the proportion of the fault data in the time window. However, the average diagnosis rate of 10 windows corresponding to the samples of faults 1 and 4 was lower than 1, and the four samples of fault 1 were diagnosed as fault 2 in the last window. The reason may be that as time goes on, the process variables become more and more affected by the failure, leading to the gradual narrowing of differences between failure types.

In order to compare the differences with other methods, the multiway dynamic kernel principal component analysis (MDKPCA) method commonly used for online inspection of the batch process is constructed in this paper to train and test the same data. The fault detection time (FDT) and false alarm rate (FPR) are calculated according the follow formulas:

(22) $$\mathrm{FDT}=T_{ide}-T_{intro},$$

(23) $$\mathrm{FPR}=\frac{FN}{TP},$$

where *T_ide_* is the time that the fault has been detected; *T_intro_* is the time of fault introduction; *FN* is the number of normal data that has been detected as fault; *TP* is the number of normal data.

The FDT and FPR results between LAS-QTA and MDKPCA are shown in Table 5. FDT of MDKPCA is higher than LAS-QTA’s according to the table. The reason is that MDKPCA cannot amplify local information based on its calculation method. It calculates the statistical parameters for the entire normal batch, and standardizes the same, which masks the minor changes of the early fault introduction. The higher false positive rate of MDKPCA may be due to the fact that the threshold of the algorithm itself is a unique value. In conclusion, this method has a better comprehensive effect in early fault detection than MDKPCA.

## 4. Conclusions

Early fault diagnosis technologies of batch processes ensure smooth operation of the chemical plant, reducing unnecessary losses. However, the data-based fault diagnosis method makes it difficult to mine the data information with a high mechanism, which can easily deviate from reality. Meanwhile, the traditional semi-quantitative method—the trend analysis method—has some problems, such as difficulties in expressing different trends and being easily affected by noise. Therefore, we proposed a new trend analysis method, based on time window adaptive standardization and Euclidean distance to extract incipient fault signal and improve the fault diagnosis result of batch processing. Adaptive normalization based on the time window enables time segments with the same trend but different values to be transformed into the same category. The data difference under the time window is enlarged. The new trend analysis also includes the function coefficients fitted by the least square method as trend information, reducing the effect of noise. In addition, the study used the different distances between classes in space to find an appropriate trend matching method referring to the basic principle of pattern recognition. Additionally, we constructed matching criteria and method. Finally, penicillin fermentation proved to be valid. To study the scalability and robustness of the method, we set the two groups of test data for fault diagnosis analysis according to whether there was corresponding normal batch data in the history database. The results show that the method is scalable. The average fault diagnosis rate is 100% and 87%, respectively. Compared with the traditional batch early fault diagnosis method MDKPCA, the fault detection time is shortened by 46 sampling points, and the false positive rate is lower than MDKPCA.

Compared with the traditional QTA method, this method can carry out online diagnosis, but compared with other data-driven methods, there is still a certain distance in fault identification. The results show that when the historical database does not contain the corresponding historical data, the robustness of the system is poor. In addition, the characteristics of batch data also include the unequal length of time between batches, which also affects the self-learning and scalability of the method, which will be the author’s next research direction. At the same time, different clustering methods for fault identification will improve the overall fault diagnosis effect. Selecting the appropriate method that combines well with LAS-QTA is another direction of future research.

## Figures and Tables

**Table 1:** The initial set values of the normal batches.

Variable Name	Unit	Set Value			
		Batch 1	Batch 2	Batch 3	Batch 4
substrate conc.	g∙L^−1^	15	14	16	14
dissolved oxygen	% saturation	1.16	1.00	1.20	1.02
carbon conc.	mol∙L^−1^	0.0005	0.0005	0.0006	0.00052
culture volume	L	100	100	100	100
temperature	K	298	298	298	298
penicillin conc.	g∙L^−1^	0	0	0	0
pH	-	5.0	4.8	5.1	4.8
biomass conc.	g∙L^−1^	0.1	0.1	0.1	0.1

**Table 2:** The details of test samples 1~12.

Variable Name	Unit	Set Value											
		S1	S2	S3	S4	S5	S6	S7	S8	S9	S10	S11	S12
fault type		aeration rate step increasing				agitator power step increasing				substrate feed rate step increasing			
magnitude	%	10	30	60	80	15	30	55	70	15	30	50	60
occurrence moment	h	80	90	100	110	111	90	150	65	80	90	70	105

**Table 3:** The details of test samples 13~24.

Variable Name	Unit	Set Value											
		S13	S14	S15	S16	S17	S18	S19	S20	S21	S22	S23	S24
fault type		aeration rate step decreasing				agitator power step decreasing				substrate feed rate step decreasing			
magnitude	%	25	30	45	65	15	30	50	70	15	32	45	75
occurrence moment	h	68	90	130	100	90	78	80	70	100	180	150	111

**Table 4:** The fault diagnosis result of LAS-QTA method.

Sample No.	Occurrence Moment (h)	Detect Moment (h)		Result		Actual Fault Type
		Batch 1	Batch 4	Batch 1	Batch 4	
**1**	80	80	80	1	1	1
**2**	90	90	90	1	1	
**3**	100	100	100	1	1	
**4**	110	110	110	1	1	
**5**	111	111.1	111.1	2	2	2
**6**	90	90.5	90.5	2	2	
**7**	150	150.3	150.3	2	2	
**8**	65	65.5	65.5	2	2	
**9**	80	80.4	80.4	3	3	3
**10**	90	90.5	90.5	3	3	
**11**	70	70.8	70.8	3	5	
**12**	105	105.1	105.1	3	5	
**13**	68	68	68	4	4	4
**14**	90	90.1	90.1	4	4	
**15**	130	130	130	4	4	
**16**	100	100	100	4	4	
**17**	90	90.7	90.7	5	3	5
**18**	78	78.1	78.1	5	5	
**19**	80	80.1	80.1	5	5	
**20**	70	70.2	70.2	5	5	
**21**	100	100.2	100.2	6	6	6
**22**	180	180.1	180.1	6	6	
**23**	150	150.2	150.2	6	6	
**24**	111	111.1	111.1	6	6	

**Table 5:** The compared result between LAS-QTA and MDKPCA.

Sample No.	FDT (h)		FPR	
	LAS-QTA	MDKPCA	LAS-QTA	MDKPCA
**1**	0.0	6.9	0.0000	0.1708
**2**	0.0	5.2	0.0000	0.2011
**3**	0.0	0.9	0.0000	0.2051
**4**	0.0	2.0	0.0000	0.2312
**5**	0.1	1.0	0.0000	0.2218
**6**	0.5	5.2	0.0000	0.1932
**7**	0.3	3.1	0.0000	0.2308
**8**	0.5	14.5	0.0000	0.2281
**9**	0.4	1.0	0.0000	0.2139
**10**	0.5	5.2	0.0000	0.2281
**11**	0.8	16.9	0.0000	0.2869
**12**	0.1	3.6	0.0000	0.3182
**13**	0.0	11.4	0.0000	0.2045
**14**	0.1	5.2	0.0000	0.1876
**15**	0.0	2.2	0.0000	0.2248
**16**	0.0	0.9	0.0000	0.2010
**17**	0.7	5.2	0.0000	0.2079
**18**	0.1	1.5	0.0000	0.1883
**19**	0.1	6.9	0.0000	0.1848
**20**	0.2	9.4	0.0000	0.2000
**21**	0.2	0.2	0.0000	0.1485
**22**	0.1	3.2	0.0000	0.2106
**23**	0.2	3.1	0.0000	0.1953
**24**	0.1	1.5	0.0000	0.1518
**mean**	0.2083	4.8042	0.0000	0.2098

## Data Availability

The study did not report any data.
